# Do bonuses affect teacher staffing and student achievement in high poverty schools? Evidence from an incentive for national board certified teachers in Washington State

**DOI:** 10.1016/j.econedurev.2018.06.010

**Published:** 2018-08

**Authors:** James Cowan, Dan Goldhaber

**Affiliations:** a American Institutes for Research, 1000 Thomas Jefferson Street NW, Washington DC 20007, USA; b Center for Education Data and Research, University of Washington, Bothell, 3876 Bridge Way N, Suite 201, Seattle, WA 98103, USA

## Abstract

We study a teacher incentive policy in Washington State that awards a financial bonus to National Board certified teachers in high poverty schools. Using a regression discontinuity design, we find that the bonus policy increased the proportion of certified teachers in bonus-eligible schools by improving hiring, increasing certification rates of incumbent teachers, and reducing turnover. Depending on the method, we estimate that the proportion of NBCTs in treated schools increased by about four to eight percentage points over the first five years of eligibility. However, the improvement in certification rates corresponds to a change of about 0.2–0.3% of a standard deviation in teacher quality per year and we do not find evidence that the bonus resulted in detectible effects on student test achievement.

## Highlights

• We study a teacher incentive policy in Washington State that awards a financial bonus to National Board certified teachers in high poverty schools.
• The policy increased the proportion of board certified teachers through improved hiring, increased certification rates, and reduced turnover.
• However, the improvement in certification rates corresponds to a change of about 0.2–0.3% of a standard deviation in teacher quality per year and we do not find evidence that the bonus resulted in detectible effects on student test achievement.

## 1 Introduction

Teacher quality is among the most variable school-based influences on student learning. Using data from a random assignment experiment, Nye, Konstantopoulos, and Hedges (2004) estimate that within-school differences in teacher quality explain about 12–14% of student achievement gains in math and about 7% of achievement gains in reading at the elementary school level. The magnitude of these findings is consistent with a large number of observational studies (Aaronson et al., 2007, Rivkin et al., 2005). Moreover, more recent research has linked teacher assignments to non-academic and distant outcomes like school attendance, educational attainment, and earnings (Chetty et al., 2014, Gershenson, 2016, Jackson, 2016, Koedel, 2008). However, as with other schooling resources, not all students have equitable access to high-quality teachers, whether measured by observable credentials or effects on student achievement (Clotfelter et al., 2005, Goldhaber et al., 2015, Lankford et al., 2002, Mansfield, 2015, Sass et al., 2012).

In response to these discrepancies, policymakers have become increasingly interested in using financial incentives to increase the number of effective teachers in high poverty and other hard-to-staff schools. In 2014, the U.S. Department of Education announced requirements for all states to develop plans for addressing inequities in the staffing of schools. These plans require states to measure inequities in the assignment of low income and minority students to effective teachers. By law, states are required to report on several observable teacher characteristics: experience, teacher qualifications (e.g., state certification), and in-field teaching credentials (Williams, Adrien, Murthy, & Pietryka, 2016). States must additionally submit plans for reducing observed inequities. A number of states proposed offering additional financial incentives, including salary bonuses, loan forgiveness, or signing bonuses, to attract teachers to low income or other high needs schools (Klein, 2015a, Klein, 2015b, Klein, 2015c, Williams et al., 2016).

There is currently limited evidence on the extent to which such targeted teacher bonuses affect student achievement in hard-to-staff schools. A number of studies have found that targeted bonuses improve teacher retention (Clotfelter et al., 2008, Springer et al., 2016). There is relatively little evidence, however, on whether additional compensation for teachers in low income schools improves either teacher hiring or students’ academic outcomes. A key finding of the teacher quality literature is that observable characteristics of teachers and their preparation typically explain little of the variation in teachers' value-added contributions to student achievement (Aaronson et al., 2007, Harris and Sass, 2011, Rivkin et al., 2005). There is also some uncertainty about whether teachers are sufficiently responsive to financial incentives to work in high-needs schools to improve student learning outcomes (Protik, Glazerman, Bruch, & Teh, 2015). It therefore remains an open question whether compensation policies targeted on teacher credentials or direct measures of productivity can substantially improve the quality of instruction in high-needs schools.

In this study, we assess an incentive policy in Washington State designed to increase the supply of effective teachers in high poverty schools. The Challenging Schools Bonus (CSB) awarded a $5,000 annual bonus to teachers who earned certification through the National Board for Professional Teaching Standards (NBPTS) and worked in schools with a high proportion of students qualifying for subsidized lunches.^1^ The CSB provides a good test of the potential for targeted incentives based on teaching credentials as it is one of the few credentials consistently linked to student achievement gains in the teacher effectiveness literature (Cavalluzzo et al., 2015, Clotfelter et al., 2007, Clotfelter et al., 2010, Cowan and Goldhaber, 2016, Goldhaber and Anthony, 2007). As with other indicators of teacher quality, NBCTs are less likely to teach in high poverty schools (Elfers and Plecki, 2014, Goldhaber et al., 2007, Humphrey et al., 2005, Sass et al., 2012). At least five other states offer additional compensation for National Board certified teachers (NBCTs) that is similar to the CSB and two states have included National Board certification status in their teacher equity reports (National Board for Professional Teaching Standards 2015, Williams et al., 2016).

We study the effects of the NBCT bonus policy in Washington using a regression discontinuity design based on the schoolwide eligibility rule. We find that eligibility for the additional compensation increased the number of NBCTs in high poverty schools by improving hiring, encouraging certification among incumbent teachers, and reducing turnover among Board certified teachers. Over the first six years of the program, we estimate that eligibility increased the proportion of NBCTs by about 0.7–1.6 percentage points per year. The largest effects operate through increases in the number of teachers earning professional certification. However, to the best of our knowledge, this is also one of the first papers to document the recruitment effects of differentiated compensation policies in education.^2^ Despite the evident improvements in teacher staffing, we do not find positive student achievement effects from the bonus policy. Based on estimates of the relative effectiveness of NBCTs at raising student achievement found in the literature, our estimated effects on school staffing imply annual improvements in student learning of less than 0.001 standard deviations per year of eligibility. Consistent with this prediction, our direct estimates of the effects of the bonus policy on student achievement are near zero and not statistically significant.

## 2 Background

In this paper, we explore the effects of a policy awarding additional compensation to teachers in high poverty schools. These differentiated compensation policies may counteract two features of the public schooling system in the United States. First, a significant proportion of school financing comes from local taxes. Wealthier school districts tend to raise more revenue for public schools and offer higher salaries to teachers (Ushomirsky & Williams, 2015). Second, within school districts, teacher salaries have traditionally followed a uniform schedule with limited differentiation in pay. If low income schools offer fewer nonpecuniary amenities and equivalent compensation as wealthier schools, teachers may be less inclined to accept job offers (Rosen, 1986). Indeed, prior research has found that teachers in low income schools, on average, are less effective and less likely to be fully qualified to teach (Goldhaber et al., 2015, Lankford et al., 2002, Sass et al., 2012). Differentiated compensation policies should therefore affect attrition rates and the composition of the applicant pool in targeted schools. In addition, the policy we focus on, which incentivizes a particular form of professional certification, may additionally affect the certification and labor supply decisions of incumbent, uncertified teachers.

By increasing the value of a teaching position in hard-to-staff schools relative to other teaching positions, targeted financial incentives should improve the retention of teachers in such schools. Research on the relationship between district salaries and mobility supports this hypothesis (Hanushek et al., 2004, Hendricks, 2014, Imazeki, 2005, Lankford et al., 2002). More relevant to the program we study, Clotfelter et al. (2008) found that a temporary North Carolina program that awarded $1,800 bonuses to math, science, and special education teachers in high poverty or low achieving schools reduced the turnover of targeted teachers by about 17%. Springer et al. (2016) assessed a pilot Tennessee program that paid $5,000 bonuses to highly-rated teachers in low-achieving schools and found that receipt of the bonus improved retention among teachers in tested grades and subjects. These findings suggest that differentiated compensation policies can affect the composition of teachers by altering who leaves high poverty schools.

A similar argument suggests that targeted incentives should improve the recruitment of new teachers into hard-to-staff schools. Better compensation may increase the likelihood that effective mobile teachers take positions in high-needs schools. Although there is little evidence about the effects of differentiated compensation policies on teacher recruitment, two studies of temporary recruitment bonuses have found that such policies increase the likelihood that teachers take positions in eligible schools. Glazerman, Protik, Teh, Bruch, and Max (2013) analyzed a policy that provided a randomized group of schools the opportunity to offer high-performing teachers $20,000 bonuses to transfer to a low-achieving school for at least two years. They found clear evidence of recruitment effects: teachers recruited to eligible positions were 43 percentage points less likely to have less than six years of experience and more than twice as likely to possess National Board certification. Steele, Murnane, and Willett (2010) studied a policy initiative in California, the Governor's Teaching Fellowship, that conditioned a $20,000 scholarship on teaching in a low-performing school for four years. As with the findings from the Teacher Transfer Initiative, they found a substantial increase in the likelihood that targeted teachers work in such schools. There is also complementary evidence from similar recruitment programs in medicine (Pathman et al., 2004, Rabinowitz et al., 1999) and law (Field, 2008).

Finally, beyond altering the incentives for already-certified teachers, incentive policies like the CSB also increase the value of obtaining particular credentials. State policy or teacher salary contracts frequently provide additional compensation to any teachers holding academic degrees, particular subject-area endorsements, or National Board certification. Although there is little evidence on how such policies affect the human capital investments or professional certification decisions of teachers, Feng and Sass (2017) have found that tuition reimbursement policies increased the likelihood that teachers in Florida earned endorsements in shortage areas. The implications of increased certification rates for high needs schools targeted by differentiated compensation policies, however, are ambiguous. Increasing professional certification among incumbent teachers may improve instructional effectiveness if they process of obtaining certification improves teaching practice. There is little evidence from student achievement data that participating in the NBPTS certification process improves practice (Chingos and Peterson, 2011, Goldhaber and Anthony, 2007, Harris and Sass, 2009), although Sato, Wei, and Darling-Hammond (2008) found that participation does improve teachers’ ability to perform the sorts of tasks that NBPTS assesses. On the other hand, teachers’ participation in the certification process, which NBPTS estimates requires 200–400 h, may disrupt student learning (Goldhaber and Anthony, 2007, Harris and Sass, 2009). Further, Goldhaber and Hansen (2009) have argued that earning professional certification provides other potential employers with an observable signal of a teachers’ effectiveness and increases the probability that effective teachers switch schools.

### 2.1 Washington's challenging schools bonus program

Washington State has awarded a salary incentive for NBCTs since the 1999–2000 school year. Initially set at 15% of salary, the state fixed the bonus at $3,500 per year in 2000 and raised it to $5,000 in 2007. At the same time, Washington introduced an additional bonus for teachers in high poverty schools. The program, called the Challenging Schools Bonus (CSB), awards an additional $5,000 to NBCTs.^3^ Following the increase in the standard bonus and the introduction of the CSB, the number of NBCTs in Washington rose substantially. During the first year of the new bonus programs, the number of new teachers earning certification increased from 489 to 922 (Plecki et al., 2010). By 2014, Washington was producing the most new NBCTs nationwide (National Board for Professional Teaching Standards, 2014a). Over the same time period, annual state spending on bonuses for NBCTs increased from $10,000,000 to $45,000,000 (Office of the Superintendent of Public Instruction, 2014).

The program targets teachers in the state's highest poverty schools based on the share of students eligible for free- or reduced-price lunch (FRL) programs. The minimum enrollment share, listed in Table 1, varies across years and school level. Initially, OSPI used two sources of data on FRL enrollment to determine school eligibility. The first of these is based on information reported in the state's administrative data system (Core Student Record System, CSRS).^4^ Districts also report counts of school enrollment and FRL participation to the state's Child Nutrition Services (CNS). During the first year of the program, schools with FRL enrollment exceeding 70% of total enrollment were eligible for the bonus. Thereafter, eligible schools were elementary schools with FRL enrollment exceeding 70% of total enrollment, middle schools with FRL enrollment exceeding 60%, and high schools with FRL enrollment exceeding 50%. Until 2011, schools were also grandfathered in based on their FRL enrollment share in the previous year. The three thresholds are quite close to the 75th percentile of school FRL enrollment share by school level, so the program targets roughly the highest poverty quartile of schools in the state.

**Table 1:** Challenging schools bonus eligibility rules.

Year	Data Source	Prior Year	Threshold		
			Elementary Schools	Middle Schools	High Schools
2007–2008	CSRS/CNS		70%	70%	70%
2008–2009	CSRS/CNS	Yes*	70%	60%	50%
2009–2010	CSRS	Yes	70%	60%	50%
2010–2011	CSRS	Yes	70%	60%	50%
2011–2012	CSRS	No	70%	60%	50%
2012–2013	CSRS	No	70%	60%	50%

*Notes:* Eligibility for Challenging Schools Bonus by school level and year. Elementary schools are defined as those with highest grade of 6th or lower. Middle schools are defined as those with a highest grade of 7th-9th grades. High schools are defined as those with a highest grade of 10th-12th. “Data source” denotes the data series used to estimate FRL enrollments. CSRS = Core Student Record System. In 2009–2010, the Comprehensive Education Data and Research System (CEDARS) replaced the CSRS; we maintain the labeling for simplicity. CNS = Child Nutrition Services report. “Prior year” indicates schools receiving the Challenging Schools Bonus in a prior year were grandfathered during the current school year. Schools serving fewer than 30 students are also excluded from eligibility unless they are the largest school at that grade level in the district.

⁎ To qualify for the Challenging Schools Bonus, school must have had 70% FRL enrollment in prior year regardless of grade level.

The introduction of the incentive policies coincided with a major effort to increase the number of NBCTs in Washington State through an increase in the standard bonus for NBCTs, conditional loans for application fees, and candidate support and networking initiatives (Elfers and Plecki, 2014, National Board for Professional Teaching Standards 2010b). Consequently, the number of NBCTs in Washington State increased substantially in both CSB-eligible and CSB-ineligible schools shortly after the introduction of the policy. Nonetheless, the proportion of NBCTs in high poverty schools relative to low poverty schools increased over this time period. In 2007, 2.7% of teachers in low poverty schools (those schools never eligible for the bonus) and 2.0% of teachers in high poverty schools (those schools ever eligible) were NBCTs. By 2013, the gap in Board certification between low- and high poverty schools had reversed, with 9.9% and 11.3%, respectively, having earned certification. These trends are consistent with the findings of Elfers and Plecki (2014) and Simpkins (2011), who document that the proportion of NBCTs and candidates for Board certification in high poverty schools increased after 2007.

**Fig. 1:**
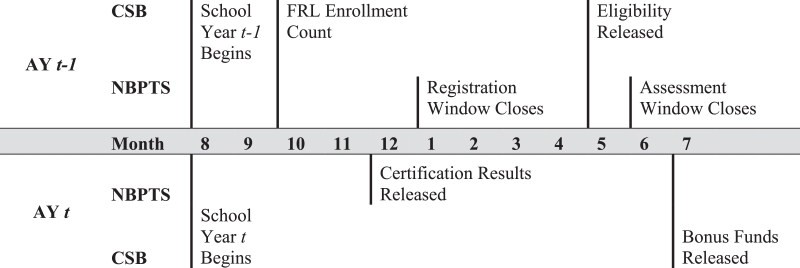
Timeline of NBPTS certification and bonus processes. *Source:*American Federation of Teachers & National Education Association 2007, American Federation of Teachers & National Education Association 2008) and National Board for Professional Teaching Standards (2010a).

To understand how the bonus policy is likely to affect teachers’ certification and labor supply decisions, it is helpful to understand the timing of the NBPTS process and CSB eligibility determination. The full process spans an approximately two year period (summarized in Fig. 1). OSPI typically determines eligibility for the upcoming school year in May or June based on enrollment data from the previous October. During the following school year, districts notify the state of eligible teachers working in their schools. Until the 2011–12 school year, Washington disbursed funds to districts after receiving these reports. Currently, the state disburses the bonus funds to school districts in the July following the conclusion of the school year and requires districts to pay out the bonus by August 31. Given the timing of eligibility determination, most staffing outcomes in a particular school year are likely related to eligibility during the specified year. As shown in Fig. 1, the state announces bonus eligibility after the NBPTS application period closes for the upcoming school year (usually on December 31 during the years of this study). Although the NBPTS assessment cycle runs through June, teachers are unlikely to know their school's eligibility in time to apply for certification before their school becomes eligible. At the same time, the announcement comes early enough that certified teachers may still participate in the job market for the upcoming school year. Teachers contemplating taking new positions during the spring or summer will generally know whether their current or potential schools will be eligible for the bonus during the coming school year. Newly certified teachers, who generally receive notification in the preceding December, should also be able to participate in the spring or summer job market.

## 3 Data and empirical methods

### 3.1 Data

In this study, we use data on student assessments and teacher staffing from two databases maintained by the Office of the Superintendent of Public Instruction (OSPI). We construct data on teacher turnover and credentials using the S-275, which is the employment reporting system for public schools in Washington State. Districts report school employees who have an employment contract in place by October 1 of each school year. Beyond school of employment, the S-275 includes information about employees' assignments and teaching credentials. These include educational attainment, experience, salary, and teacher demographics. Using this database, we construct a panel of teachers for the school years 2001–2002 through 2012–2013. We define teachers as those with base contracts that have assignments in classroom teaching positions and assign teachers to a school if their reported time in that building exceeds 50% of their total full-time equivalency.^5^ Using this assignment rule, we include all teachers working in Washington public schools that qualify to be considered for CSB eligibility.^6^ The remaining sample represents about 90% of public school employees who are reported as working in classroom teaching positions.

As we are primarily interested in how the bonus policy affects teacher retention, we define a teacher as moving if she is not assigned to the same school in the S-275 during the following academic year.^7^ This includes teachers who switch schools and those who exit the public school system. Teachers earn the bonus if they do not leave their school at the end of the year it becomes eligible. Hence, we match the mobility data to a school's eligibility in the upcoming school year.

We supplement the S-275 with data on National Board candidates provided by NBPTS. This data includes the year of submission and assessment results of all applications originating from public or private schools in Washington State. Our measure of National Board certification therefore only includes teachers who initially obtained certification in Washington and may understate the total number of NBCTs. To the extent that the bonus policy incentivizes cross-state migration to eligible schools, our estimates of staffing effects would likely be downward biased. However, cross-state teacher mobility appears to be relatively rare. During the 2011–2012 school year, while nearly 7% of teachers had worked in a different public school in the same state during the previous year, only 0.5% of teachers had worked in a public school in another state (National Center for Education Statistics, 2015). There are additionally very few NBCTs in Oregon and Idaho, the two states that share a border with Washington (National Board for Professional Teaching Standards, 2014b). Given the relative rarity of both cross-state mobility and National Board certification, the degree of measurement error in our NBCT indicator is likely to be small.

In most cases, we do not include individual level characteristics in our models. Individual teacher characteristics may be endogenous to bonus eligibility and therefore should be excluded. We therefore use data averaged at the school level in our analysis. We present summary statistics for teachers across all years of our sample in Table 2. Eligible schools constitute about 22% of the schools in our sample. In CSB schools, 14.1% of teachers depart each year, which is fairly similar to the statewide average of 12.9%. However, we do find some differences in observable teacher credentials: CSB teachers have about an average of about one year less teaching experience and are about 3 percentage points less likely to have an advanced degree. As a first indication that the CSB program may influence the supply of teachers, we find that about 8% of CSB teachers in our sample possess an NBPTS teaching certificate, compared to the sample average of 6%.

**Table 2:** Summary statistics.

	Full Sample			CSB Schools		
	Mean	SD	N	Mean	SD	N
*Panel A. Teacher characteristics*						
NBCT	0.063	0.072	10,188	0.076	0.080	2,245
NBCT candidate	0.018	0.034	10,188	0.024	0.043	2,245
Salary	60,470.61	6,090.10	10,188	60,528.07	6,664.22	2,245
Experience	13.485	2.777	10,188	12.506	2.862	2,245
Leave school	0.129	0.082	10,188	0.141	0.094	2,245
New to school	0.128	0.086	10,188	0.143	0.098	2,245
Advanced degree	0.656	0.113	10,188	0.624	0.121	2,245
*Panel B. Student Characteristics*						
Math test	0.010	0.397	28,701	−0.369	0.320	6,357
Reading test	0.006	0.350	29,655	−0.372	0.282	6,686
Asian/Pacific Islander	0.087	0.095	29,664	0.081	0.113	6,689
Black	0.050	0.074	29,664	0.081	0.119	6,689
Hispanic	0.178	0.198	29,664	0.430	0.292	6,689
White	0.624	0.227	29,664	0.344	0.239	6,689
Gifted	0.053	0.102	29,664	0.037	0.090	6,689
Limited English proficiency	0.059	0.095	29,664	0.162	0.151	6,689
Special education services	0.084	0.053	29,664	0.089	0.056	6,689
Free/reduced-price lunch	0.448	0.240	29,664	0.784	0.139	6,689

*Notes:* Summary statistics for teacher and student observations. Observations are at the school-year (for teachers) and school-grade-year level (for students). Summary statistics are for all observations between 2008 and 2013. Observations for the full analysis sample are displayed under “all schools” and for currently-eligible CSB schools under “CSB schools.”

The student-level data includes student demographic information, annual math and reading assessment data in grades 3–8 and 10, and information on participation in various special programs. Because our identification strategy ought to provide unbiased estimates of treatment effects without relying on pre-test information, we use all years for which testing outcomes are available. For 2008–2013, we use standardized reading assessments in grades 3–8 and 10 and standardized math assessments in grades 3–8. In 2010, the state replaced the 10th grade standardized math assessment with end-of-course assessments in algebra and geometry. Given that the decisions about the timing of end-of-course assessments may be endogenous to bonus eligibility, we only consider outcomes on the standardized 10th grade math assessment from 2008–2010. We standardize all assessments by grade and year. We present summary statistics for students in Panel B of Table 2. Unsurprisingly, CSB-eligible schools are quite different than the state as a whole. Student achievement is nearly 0.4 standard deviations lower in CSB schools than in other schools during the same time period. Students are also much more likely to be members of an underrepresented minority: 43% and 8% of students in CSB schools are Hispanic and African-American, respectively, compared to just 17% and 5% overall. Not surprisingly given these demographics, CSB students are nearly three times more likely to participate in bilingual programs than the state average.

### 3.2 Research design

To identify the effect of the bonus policy on student achievement and teacher staffing, we exploit the discontinuous relationship between school-wide FRL enrollment share and bonus eligibility using a regression discontinuity design (RDD). The RDD relies on the fact that schools with comparable poverty levels near the eligibility thresholds fall into different treatment states. By focusing on changes in outcomes at the eligibility threshold, the regression discontinuity approach ignores variation in outcomes that may be associated with factors correlated with school poverty but not caused by the program itself. For instance, the introduction of the bonus policy coincided with an increase in the standard bonus for NBCTs in Washington and several rounds of teacher layoffs during the recession; both of these factors likely have disparate effects in high and low poverty schools (Goldhaber, Strunk, Brown, & Knight, 2016).

Previous research has suggested that schools react strategically to discontinuous eligibility rules. For instance, in an analysis of a large Northeastern school district, Matsudaira, Hosek, and Walsh (2012) found that schools clustered just above the eligibility threshold for school-wide Title I funding, and that this clustering followed changes in the eligibility threshold. Such manipulation of program eligibility potentially undermines the RDD. If schools can adjust their enrollment in order to qualify for the bonus program, then the treatment status of schools near the eligibility threshold may no longer be independent of a school's counterfactual outcomes. In the context of the CSB policy, schools with a greater baseline share of NBCTs might face greater incentives to boost their FRL enrollment in order to qualify for the CSB, which would bias our estimates upward.

As in prior research using schoolwide eligibility rules, we find that schools are significantly more likely to barely qualify for the CSB. The eligibility rule described in Table 1 is a complicated function of two series of FRL eligibility data. Let *FRL^CSRS^* denote the CSRS poverty measure, *FRL^CNS^* denote the CNS poverty measure, *FRL* = max(*FRL^CSRS^, FRL^CNS^*) be the maximum of the two school FRL enrollment shares, and *c* be the school-specific threshold in Table 1. Then define

(1) $$x_{st}=\left\{ \begin{array}{cc} FRL_{st}-0.7 & \text{if}\;t=2008\\ \max\left(FRL_{st}-c_{st},\;FRL_{st-1}-c_{st-1}\right) & \text{if}\;t=2009\\ \max\left(FRL_{st}^{CSRS}-c_{st},\;FRL_{st-1}-c_{st-1}\right) & \text{if}\;t=2010\\ \max\left(FRL_{st}^{CSRS}-c_{st},\;FRL_{st-1}^{CSRS}-c_{st-1},\;FRL_{st-2}-c_{st-2}\right) & \text{if}\;t=2011\\ FRL_{st}^{CSRS}-c_{st} & \text{if}\;t\geq \;2012 \end{array}\right.$$

Schools are eligible for the CSB if and only if *x_st_* ≥ 0. We estimate the discontinuity in the density of this forcing variable in Table 3.^8^ We find a statistically significant discontinuity in the density of 0.210, suggesting 21% more observations to the right of the eligibility threshold than expected. In column 2, we ignore the grandfathering provision and estimate the discontinuity in the maximum of the two poverty measures from the prior year only. The estimated discontinuity is 0.081 and not statistically significant. The fact that the discontinuity in the eligibility rule is larger indicates that it is not the same programs that just exceed the eligibility threshold in each year.

**Table 3:** Discontinuities in the density of the forcing variable.

	Forcing Variable	
	Eligibility Rule	Current enrollment
Both counts	0.2102⁎⁎	0.0814
	(0.0947)	(0.0759)
N	10,188	10,188
CSRS FRL Count	0.0871	0.0161
	(0.0922)	(0.0760)
N	10,188	10,188
CNS FRL Count	0.3092*	0.1731
	(0.1643)	(0.1580)
N	6,792	3,396

*Notes:* We estimate the discontinuity in the forcing variable using the method of McCrary, 2008a, McCrary, 2008b). The both counts measure is constructed using the full set of poverty measures considered by OSPI in the relevant year (see Table 1 for details). The CSRS count uses the FRL count from the CSRS data only. The CNS count uses the FRL count from the CNS data for 2008–2009 for eligibility during the 2008–2011 school years (column 1) and 2008–2009 school years (column 2). The eligibility rule column uses the true eligibility rule for the relevant year, including historical data where appropriate, while the current enrollment column uses the current year's enrollment only. The standard errors are estimated using a school-level block bootstrap with 400 iterations.

⁎ *p* < 0.10,

⁎⁎ *p* < 0.05, ^⁎⁎⁎^*p* < 0.01.

The true eligibility rule uses two sources of data on FRL enrollment. One of these (CNS) is a survey completed by school administrators, while the other (CSRS) is based on administrative enrollment records. In the remaining rows, we show that the discontinuity in the forcing variable is largely attributable to the survey measure. In the first column, we reconstruct an alternative version of the forcing variable using only one of the sources of poverty data. This alternative measure uses the historical data in the same way, but omits the other poverty count. We then construct a contemporaneous measure using the only the prior year's poverty share. In the second row, we find little evidence of manipulation in the CSRS poverty count. The discontinuity in the forcing variable is 0.087 and not statistically significant; it is 0.016 when looking at the contemporaneous measure only. The discontinuities in the CNS measure are much larger, although they are used in fewer years and are therefore less precisely estimated. In the first column, we consider the CNS eligibility rule for 2008–2011. OSPI stopped using the contemporaneous CNS measure in 2010, but the grandfathering provisions ensured that historical CNS values were still used in determining eligibility through 2011. We estimate a discontinuity in this variable of 0.309, which is statistically significant at the 10% level. In the second column, we show the discontinuity in the contemporaneous measure for 2008 and 2009, the two years in which it was used to determine eligibility. The discontinuity of 0.173 is smaller than in the first column and statistically insignificant, but much larger than the discontinuity in the CSRS measure (0.016).

Given the evidence for manipulation in the CNS measure, we use only the CSRS count for the remainder of the analysis. We construct an analog of the eligibility rule using the CSRS data as in row 2, column 1 of Table 3; that is, we replace *FRL^CSRS^* for *FRL* in Eq. (1) above. We then implement the RDD using only this CSRS eligibility measure as a forcing variable. Because some schools qualify for the CSB without having a CSRS poverty count that exceeds the relevant threshold, this describes a fuzzy regression discontinuity. Using the forcing variable *x^CSRS^*, we estimate

(2) $$\begin{array}{c} Y_{st}=\delta CSB_{st}+f\left(x_{st}^{CSRS}\right)+\epsilon_{st}\\ CSB_{st}=\gamma 1\left(x_{st}^{CSRS}\geq 0\right)+g\left(x_{st}^{CSRS}\right)+\eta_{st} \end{array}$$

using school-year aggregates of the teacher data and school-grade-year aggregates of the student data for 2008–2013. The one exception is the teacher mobility outcome, which should be related to the next school year's eligibility. In this case, we replace *x^CSRS^* and *CSB* with their leads. We estimate *f()* by local linear regression with a triangular kernel and weighting by the number of observations in each school-year cell.^9^ We estimate Eq. (2) using the optimal bandwidths for discontinuities in each of the outcome measures suggested by Imbens and Kalyanaraman (2012) for the grouped data, which are generally about 0.35–0.40. In practice, we implement the regression discontinuity by weighted two-stage least squares. In addition to the baseline specification in Eq. (2), we estimate two variations of the RDD. First, because we pool data across several years and because the eligibility rules changed over time, the difference in treatment duration between just-eligible and just-ineligible schools varied by year. Between 2008 and 2010, exceeding the eligibility threshold in one year guaranteed at least two years of CSB eligibility. We therefore instrument the number of years of cumulative eligibility, rather than current eligibility status, with the indicator for whether the school exceeds the threshold poverty level. Second, to exploit the panel nature of the data, we estimate models that include a number of school covariates, including all of the outcome measures from the year prior to the implementation of the CSB program. These include school grade level, school racial composition, current FRL enrollment share, log enrollment, 2007 mean student achievement in math and reading, and mean 2007 teacher NBCT status, experience, advanced degree possession, and turnover.

The RDD provides an estimate of a local average treatment effect (LATE) of CSB eligibility for schools near the eligibility threshold. The effect of the bonus policy on teacher staffing may differ between schools near the eligibility threshold and those with higher poverty for two primary reasons. First, the probability of future bonus eligibility is lowest for schools that are just eligible in the current year. Relative to schools farther from the eligibility threshold, the value of job offers to NBCTs and earning professional certification should be less in just-eligible schools. For instance, the discounted value of a job offer to an NBCT from a just-eligible school exceeds that of a just-ineligible school by $7,250 between 2008 and 2010, by $8,601 in 2011, and by $5,000 in 2012 to 2013.^10^ This value may be significantly larger in schools with substantially higher poverty shares: beginning in 2012, over a ten year horizon, the discounted value of an offer in a school guaranteed to receive the bonus exceeds an offer to a just-ineligible school by $21,881. By the same token, there are weaker incentives for certification in just-eligible schools. Moreover, because teachers only receive 60% of the value of the bonus during their first year of certification, the financial value of certification is smaller than the value of a job offer to a previously certified teacher. The effects of the bonus may be further moderated by the district poverty shares. Teachers may take into account the number of other nearby schools that offer the bonus when deciding whether to earn certification. To the extent that schools near the threshold are in higher income school districts than those with higher poverty levels, they may have fewer nearby schools that are eligible for the CSB. On the other hand, the value of nonpecuniary amenities may be highest near the eligibility threshold. Teachers therefore face a likely tradeoff between greater certainty surrounding bonus eligibility and other attributes of jobs in high poverty schools. The relationship between the effectiveness of the bonus policy and student poverty, and hence the relationship between the LATE we estimate and the average effect of the policy on treated schools, is thus ambiguous. We return to the external validity of our findings in Sections 4.2 and 4.3.

### 3.3 Testing the identifying assumptions of the RDD

**Fig. 2:**
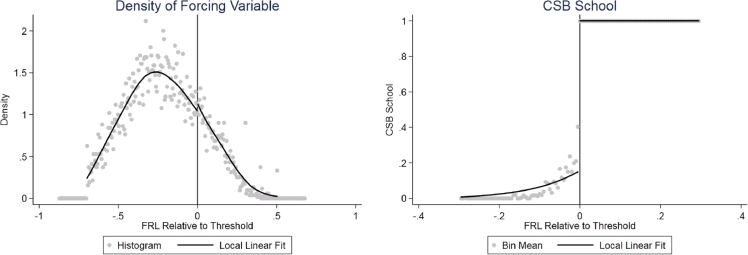
Density and Treatment Discontinuities at the Eligibility Threshold. *Notes:* In the left panel, we estimate the discontinuity in the forcing variable using the method of McCrary, 2008a, McCrary, 2008b). The scatter plot indicates the empirical density for bins of width of 0.005. We estimate a discontinuity of 0.087 (s.e. = 0.092). The standard error is estimated using a school-level block bootstrap with 400 iterations. In the right panel, we estimate the probability that a school is eligible for the bonus. The scatter plot indicates mean eligibility for cells of width 0.005 weighted by the number of teachers in each school. The line plot is the local linear conditional expectation using a triangular kernel, teacher observation weights, and a bandwidth of 0.34 (the Imbens and Kalyanaraman (2012) optimal bandwidth for the NBCT outcome).

We briefly describe the relationship between the forcing variable and school eligibility in Fig. 2. We first plot the density of the forcing variable and its relationship with CSB eligibility. The discontinuity at the eligibility threshold, which corresponds to the estimate in Table 3, is 0.087 and not statistically significant. The forcing variable also predicts eligibility for the bonus (right panel). Because eligibility is based on the maximum of the CSRS and CNS measures, every school to the right and some portion of schools to the left of the threshold receive the bonus. The first stage estimate of the discontinuity in eligibility status is 0.845 with a t-statistic of 42.6. However, the just-eligible schools include some whose eligibility is driven by their poverty counts in prior years. Thus, on average, those schools just eligible for the CSB have been eligible for about 1.5 years longer than those just ineligible.^11^

We next turn to an analysis of discontinuities in pre-treatment outcomes and student characteristics at the eligibility threshold. In Table 4, we first investigate whether there are differences in the composition in school enrollment at the eligibility threshold. Schools just to the right of the eligibility threshold have statistically significantly higher enrollments of African American and Asian students. None of the other discontinuities is statistically significant. Using the school-level data, we predict average achievement using the remaining student characteristics included in the table. The estimated discontinuities in math and reading achievement are both about −0.01 and not statistically significant. An additional concern is that school choice may be related to eligibility for the CSB program. Staffing improvements at eligible schools may lead to improvements in student retention and differentially affect the composition of test takers in eligible schools near the threshold. The attrition and mobility results in Table 4 suggest this is unlikely to be a problem in our study. We uncover little evidence of selective attrition. Point estimates in math and reading are 0.5 and −0.02 percentage points, respectively, and are not statistically significant. Similarly, the point estimate on student mobility is 0.3 percentage points and not statistically significant. Overall, we fail to reject the null hypothesis that the discontinuities in student characteristics at the eligibility threshold are jointly zero.

**Table 4:** Test of covariate balance near eligibility threshold.

Male	0.1249	Free- or reduced-price lunch	0.4658
	(0.2160)		(0.7125)
Asian/Pacific Islander	1.7868⁎⁎	Student attrition (math)	0.4416
	(0.7280)		(0.5346)
Black	2.1471⁎⁎⁎	Student attrition (reading)	−0.0149
	(0.7355)		(0.4739)
Hispanic	0.1735	Student mobility	0.3876
	(1.2897)		(1.5943)
Gifted	−0.0172	Predicted math achievement	−0.0093
	(0.9301)		(0.0115)
Limited English proficient	0.2710	Predicted reading achievement	−0.0124
	(0.6187)		(0.0105)
Special education	0.3287		
	(0.2993)		
	0.1249		
*N*			19,958
Test of joint significance (p-value)			0.2117

*Notes:* Student characteristics are the contemporaneous values of student demographic and program participation variables. Discontinuities are estimated by local linear regression with a bandwidth of 0.35. Standard errors clustered by school are in parentheses.

*p < 0.10,

⁎⁎ *p* < 0.05,

⁎⁎⁎ *p* < 0.01.

In Table 5 we test for differences in pre-treatment outcomes using teacher and student data between 2003 and 2007. We estimate these models using the 2008–2013 forcing variable and eligibility data and the 2003–2007 outcomes. The coefficients therefore describe the discontinuities in baseline outcomes at the actual eligibility thresholds. Just-eligible schools tend to have fewer NBCTs prior to the adoption of the CSB program. The discontinuity for the year before implementation of the CSB, 2007, is −0.4 percentage points and statistically significant. The remaining staffing outcomes are also generally worse on the right side of the eligibility threshold. The full set of discontinuities in Panel A are not jointly statistically significant over the full pre-treatment period, but they are statistically significant in 2007, the last year before the introduction of the bonus program.^12^ In Panel B, we test for discontinuities in the student achievement outcomes. Before 2006, Washington only tested in grades 4, 7, and 10. We therefore use the annual testing data in the two years before the implementation of the CSB for which end-of-grade tests are available for the same grades used in the main analysis. The estimated discontinuities are small and not statistically significant.

**Table 5:** Discontinuities in pre-treatment outcomes.

	2003	2004	2005	2006	2007
*Panel A. Staffing outcomes*					
NBCT	−0.0003	−0.0008	−0.0019*	−0.0020	−0.0043⁎⁎
	(0.0006)	(0.0008)	(0.0010)	(0.0016)	(0.0022)
NBCT Applicant	−0.0000	−0.0011	−0.0015	0.0008	−0.0024*
	(0.0009)	(0.0014)	(0.0017)	(0.0013)	(0.0013)
Experience	−0.4373⁎⁎	−0.2124	−0.2371	−0.1738	−0.2087
	(0.1907)	(0.1875)	(0.1939)	(0.1989)	(0.2066)
Advanced degree	−0.0241⁎⁎	−0.0192⁎⁎	−0.0172*	−0.0172⁎⁎	−0.0133
	(0.0097)	(0.0093)	(0.0093)	(0.0086)	(0.0083)
Salary	−228.81	111.94	108.31	105.92	156.94
	(285.78)	(284.66)	(283.92)	(297.25)	(348.56)
Turnover	0.0023	0.0038	−0.0072	0.0128⁎⁎	0.0015
	(0.0059)	(0.0060)	(0.0113)	(0.0062)	(0.0061)
*N*	6,514	6,708	6,753	6,643	6,877
Test of joint significance (*p*-value)	0.0610	0.1961	0.1068	0.1241	0.0473
*Panel B. Student Outcomes*					
Math test				−0.0012	−0.0107
				(0.0176)	(0.0178)
Reading test				0.0020	−0.0052
				(0.0173)	(0.0176)
					
*N*				18,913	19,169
Test of joint significance (p-value)				0.9596	0.7778

*Notes:* Pre-treatment outcomes give 2003–2007 values (by column) of key student and teacher variables for values of the running variable between 2008 and 2013. Discontinuities are estimated by local linear regression with a bandwidth of 0.34. Standard errors clustered by school are in parentheses.

⁎ *p* < 0.10,

⁎⁎ *p* < 0.05, ^⁎⁎⁎^*p* < 0.01.

The analysis of pre-treatment outcomes and student characteristics around the eligibility threshold may cast some doubt on the validity of the regression discontinuity design. In the analyses that follow, we take some steps to mitigate any potential bias from non-random sorting. In particular, we estimate models that include controls for all of the pre-treatment outcomes so that identification comes from changes in staffing and student achievement during the CSB period. We also show that the results are not sensitive to excluding schools very close to the eligibility threshold. Nonetheless, we argue that the patterns observed in Tables 4 and 5 indicate that our estimates of staffing effects likely provide a lower bound on the true treatment effect. Eligible schools tend to have worse pre-treatment staffing outcomes conditional on school poverty, which suggests that they are likely to have worse staffing outcomes during the CSB period as well. Although our estimates may be upwardly biased if schools that expect to hire more NBCTs in the future are able to manipulate their FRL enrollment to become eligible, the estimates in Table 5 do not suggest this is the case. There is no apparent upward trend in the discontinuities in the proportion of NBCTs at the eligibility threshold between 2003 and 2007. In fact, the point estimates are slightly more negative in 2007 than in earlier years. Furthermore, teachers in just-eligible schools are actually less likely to have applied for certification during 2007 and we do not observe any effects on any non-incentivized staffing outcomes (such as experience or educational attainment).

## 4 Results

### 4.1 Certification bonuses, school staffing, and student achievement

**Fig. 3:**
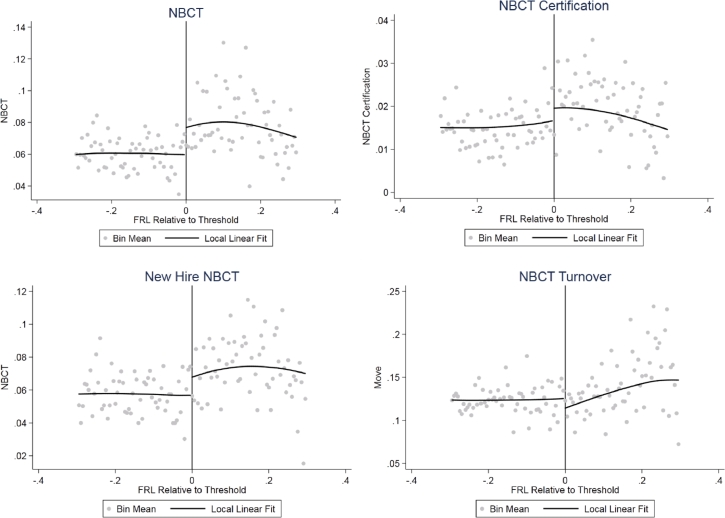
Regression Discontinuity Estimates of Staffing Effects. *Notes:* Scatter plot indicates mean eligibility for cells of 0.005 weighted by the number of teachers in each school. Line plot indicates local linear conditional expectation using a triangular kernel and weighting by the number of teachers in each school. For panels 2–4, the samples use only the relevant samples of teachers: teachers without professional certification (newly certified NBCTs), newly hired teachers (newly hired NBCTs), and NBCTs (NBCT turnover) with weights based on the size of the sample at the school level. The bandwidths are estimated using the procedure of Imbens and Kalyanaraman (2012). The bandwidths for each outcome are as follows: NBCT (0.34), newly certified NBCTs (0.45), newly hired NBCT (0.40), and NBCT turnover (0.40).

We begin our presentation of the results with graphical evidence from the regression discontinuity designs. The basic empirical results are summarized in Fig. 3. In the first panel, we show a discontinuity in the share of NBCTs of about 1.7 percentage points at the eligibility threshold. In the following panels, we show that there are also discontinuities in the proportion of non-NBCTs earning certification, the proportion of NBCTs among newly hired teachers, and the turnover rate among incumbent NBCTs. Each year, non-NBCTs in just-eligible schools are 0.6 percentage points more likely to earn certification. Among newly hired teachers, those in just-eligible schools are about 0.9% more likely to be NBCTs. Finally, NBCTs in schools that are just eligible for the bonus in the upcoming school year are 2.7 percentage points less likely to leave their schools at the end of the year.

We present more formal estimates for the NBCT staffing outcome in Table 6 that account for differences in eligibility at the threshold. We include the fuzzy regression discontinuity estimates in the first two columns, but focus our discussion on the results that use eligibility to instrument for cumulative treatment in the second two columns given the likely dynamic effects of the bonus program. Recall that changes to the grandfathering provision affect the length of eligibility guaranteed to schools that exceed the threshold. The IV estimators scale the reduced-form treatment effects underlying the estimation of the simple RD by the discontinuity in the length of treatment at the eligibility threshold rather than by the difference in the probability of current treatment. These models therefore provide an estimate of the effect of an additional year of CSB eligibility. Our baseline estimates suggest that eligibility increased certification by about 1.2 percentage points per year of eligibility. In the final column, we include pre-treatment outcomes as additional controls. As expected given the lower 2007 certification levels at the eligibility threshold, the inclusion of pre-treatment outcomes and other controls increases the estimated effect to about 1.7 percentage points per year of eligibility. Among schools that are just ineligible for the bonus, about 6% of teachers are NBCTs, so this represents an increase of about 20–28% per year of eligibility. Put another way, the mean number of teachers in our sample is about 29.5, which suggests an overall increase of about 0.3 NBCTs per year.

**Table 6:** Effect of the challenging schools bonus on proportion of board certificated teachers.

	Current Eligibility		Cumulative Eligibility	
	(1)	(2)	(3)	(4)
NBCT	0.0202⁎⁎⁎	0.0280⁎⁎⁎	0.0115⁎⁎⁎	0.0164⁎⁎⁎
	(0.0068)	(0.0054)	(0.0039)	(0.0032)
*N*	6,877	6,789	6,877	6,789
Covariates	N	Y	N	Y

*Notes:* The regression discontinuity models are estimated by two-stage least squares with triangular kernel and observation weights. The bandwidth of 0.34 is estimated using the procedure of Imbens and Kalyanaraman (2012). Covariates include 2007 school values of the proportion of NBCTs, proportion of NBCT applicants, average teacher experience, average teacher educational attainment, average teacher turnover, math and reading achievement, student race/ethnicity, student FRL participation, and school year and school level effects. Standard errors clustered by school are in parentheses.

⁎ *p* < 0.10, ^⁎⁎^*p* < 0.05, ^⁎⁎⁎^*p* < 0.01.

As we discussed above, the increase in the proportion of NBCTs could operate through any of three mechanisms: the bonus may increase the arrival rate of new NBCTs, it may increase the rate at which incumbent teachers earn certification, or it may reduce the departure rate of certified teachers. We separately consider each of these potential mechanisms in Table 7 by estimating regression discontinuity models using the relevant samples of teachers for each outcome. It is important to note that the RDD estimates effects on changes in the school average certification and mobility rates. If eligibility for the bonus affects the composition of a school's potential hires, then these effects are not necessarily the same as those for individual teachers. For instance, a school's eligibility may attract teachers who would be more likely to apply for certification even in the absence of the bonus. Increases in the certification rate or decreases in the turnover rate could therefore represent compositional changes in a school's teaching staff caused by eligibility for the bonus rather than effects on the certification or turnover propensities of individual teachers. We therefore view these results as explorations of the mechanisms underlying the staffing changes rather than estimates of the effects of incentives on individual teacher behavior. Although the former are relevant to our context because the policy targets whole schools, these effects may not generalize to the effects of system-wide retention incentives for NBCTs. In particular, the effects we estimate likely overstate the effects of such statewide policies.

**Table 7:** Effects on certification rates, hiring, and retention.

Outcome	Sample	(1)	(2)
NBPTS Certification	Non-NBCTs	0.0072⁎⁎⁎	0.0074⁎⁎⁎
		(0.0023)	(0.0022)
*N*		8,409	8,300
NBCT	New Hires	0.0101*	0.0115⁎⁎
		(0.0060)	(0.0057)
*N*		7,081	6,997
Turnover	NBCTs	−0.0321⁎⁎	−0.0424⁎⁎⁎
		(0.0142)	(0.0137)
*N*		4,041	3,970
Covariates		N	Y

*Notes:* The regression discontinuity models are estimated by two-stage least squares with triangular kernel and observation weights. The bandwidths are estimated using the procedure of Imbens and Kalyanaraman (2012). The samples are as listed in the second column and weight are determined based on the number of teachers in each sample in each school and year. The bandwidths for each outcome are as follows: NBPTS application (0.38), newly certified NBCT (0.45), newly hired NBCT (0.40), and NBCT turnover (0.40). Covariates include 2007 school values of the proportion of NBCTs, proportion of NBCT applicants, average teacher experience, average teacher educational attainment, average teacher turnover, math and reading achievement, student race/ethnicity, student FRL participation, and school year and school level effects. Standard errors clustered by school are in parentheses.

⁎ *p* < 0.10,

⁎⁎ *p* < 0.05,

⁎⁎⁎ *p* < 0.01.

In Table 7, we find evidence that the CSB program improved outcomes through all three mechanisms. Because these outcomes influence the rate of increase of NBCTs, we focus on the current eligibility indicator rather than cumulative eligibility. In the first column, we estimate the effect of eligibility for the CSB program on the certification rates for previously uncertified teachers. The certification rates in treated schools are about 0.7 percentage points higher than in untreated schools, which represents an increase in the certification rate of about 42% compared to just-ineligible schools. In column 2, we restrict the sample to newly hired teachers and estimate that the CSB program increased the proportion of newly hired teachers who were NBCTs by about 1.0 to 1.2 percentage points, or by about 38%. Finally, in column 4, we restrict the sample to current NBCTs and estimate the likelihood that a teacher leaves her school when it is eligible for the CSB in the following school year. We find a reduction of 3.2 to 4.2 percentage points, depending on the specification, which corresponds to approximately 31–41% lower turnover rates among NBCTs. During the period of our study, the average salary for NBCTs was $69,374 in just-ineligible schools. The bonus therefore represents an approximately 7.2% increase in salary, which implies a turnover elasticity of between −4.3 and −5.7. The reduction in turnover is similar to the elasticity of about −4 found in the Clotfelter et al. (2008) assessment of a retention bonus program in North Carolina. This is notably larger than those typically found in the literature on cross-district variation in teacher salaries (e.g., Hendricks, 2014), although these policies differ from pure salary differentials across districts in at least two important respects. First, differentiated compensation policies may affect within-district teacher mobility, which is likely less costly to teachers. Second, bonuses announced by the state may be more salient to teachers than differences in district salary schedules.

Much of the existing literature on merit pay and certification bonuses assesses the impact on teacher retention. Our results suggest that hiring and the certification of incumbent teachers are empirically important components of these policies. In order to provide a rough accounting of the contribution of these factors to the overall certification rate, we decompose the change in certification rates into portions attributable to each of the three mechanisms. In particular, we multiply the effects on the conditional rates of NBCT status given a teacher is new to the school, that a teacher becomes certified given that she is not already, and on turnover given that a teacher is an NBCT in Table 7 by the probability of teachers belonging to each of these groups. We use the regression discontinuity estimates of treatment effects for each the conditional probabilities and estimate the proportions of new and certified teachers using similar local linear regressions. Recall that we estimate that the bonus increases the proportion of NBCTs by about 1.6 percentage points per year. Of this increase, we estimate that newly hired NBCTs contribute about 0.2 percentage points, newly certified teachers contribute about 0.7 percentage points, and reductions in turnover contribute 0.3 percentage points. Given differences in the estimation sample, the suggested increases in certified teachers do not perfectly match the estimates in Table 6, but the individual estimates do explain about 70% of the estimated annual increase in NBCTs.^13^ Consequently, much of the increase in certification appears to come from teachers already working in high poverty schools.

**Fig. 4:**
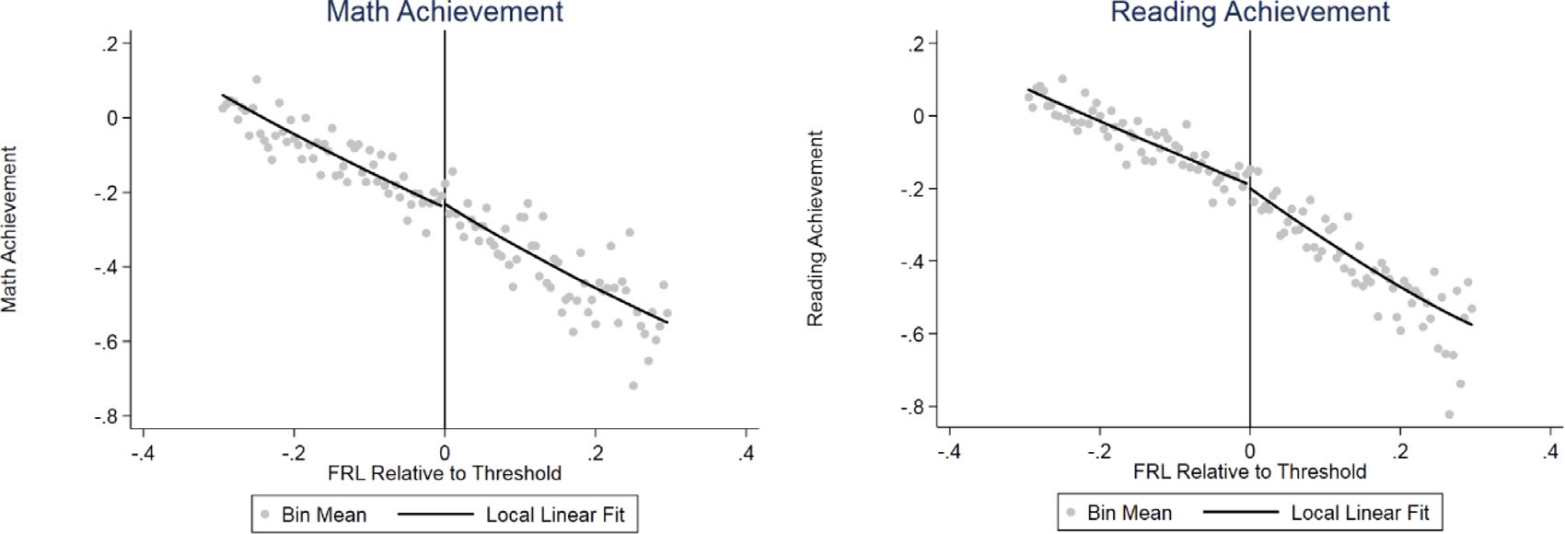
Regression discontinuity estimates of student achievement effects. *Notes:* Scatter plot indicates mean eligibility for cells of 0.005 weighted by the number of students in each school-grade. Line plot indicates local linear conditional expectation using a triangular kernel and weighting by the number of students in each school-grade cell. The bandwidths are estimated using the procedure of Imbens and Kalyanaraman (2012) (math achievement, 0.41; reading achievement, 0.35).

The literature on NBCTs suggests that they improve student achievement by about 0.01 to 0.05 standard deviations relative to non-NBCTs (Cavalluzzo et al., 2015, Clotfelter et al., 2007, Clotfelter et al., 2010, Cowan and Goldhaber, 2016, Goldhaber and Anthony, 2007). Our staffing results therefore suggest that eligibility for the bonus policy increased average student achievement by about 0.0004–0.0005 standard deviations per year, although excluding the certification of incumbent teachers reduces this amount significantly. Assuming a standard deviation of teacher effectiveness is equivalent to about 0.2 standard deviations in student achievement (Goldhaber, Liddle, Theobald, & Walch, 2012), these estimates correspond to an increase of about 0.2 to 0.3% of a standard deviation of teacher value added per year. In Fig. 4, we test the achievement effects directly using student test scores in grades 3 through 8 and 10. There is little indication in the plots of any discontinuity in test scores. We estimate discontinuities of 0.010 and −0.009 standard deviations in math and reading, respectively, although neither of these estimates is statistically significant. The formal analyses are consistent with the graphical evidence: the baseline RDD point estimates suggest that eligibility increases student achievement in math by about 0.006 standard deviations and reduces achievement by 0.006 standard deviations in reading per year, but neither of these results is statistically significant. The inclusion of covariates, including pre-treatment test scores, reduces the estimates in magnitude to 0.001 and −0.003. In both cases, the point estimates are near zero, although they are not very precisely estimated; the 95% confidence intervals exclude annual increases of about 0.032 standard deviations in math and 0.013 standard deviations in reading.

Taken together, our results suggest that the CSB policy increased the proportion of NBCTs in high poverty schools during the first five years of implementation, although the certification of incumbent teachers explains about half of the effect. We do not, however, find evidence of positive effects on student achievement. Based on previous findings on the productivity of NBCTs, any effects operating through direct instructional effects are likely to be at most 0.3% of a standard deviation in teacher quality per year of eligibility.

### 4.2 Dynamic effects of bonus policies

In Section 4.1, we estimate a LATE of CSB eligibility using an RDD. The research design implicitly uses schools near the threshold to estimate the effect of an additional year of eligibility for the bonus. This effect may differ from the average effect on treated schools for two reasons. First, schools near the eligibility threshold face greater uncertainty about the likelihood of eligibility in future years, which reduces the financial value of the bonus to teachers considering whether to apply for the bonus or transfer schools. Thus, all else equal, we should observe a weaker response in schools near the eligibility threshold. On the other hand, if teachers value working in schools with fewer low income students or other characteristics of schools that may be correlated with student income level, then schools near the eligibility threshold offer greater nonpecuniary value to teachers than schools with higher FRL eligibility shares. Thus, teachers should be more likely to switch to schools near the eligibility threshold than to schools with higher levels of student poverty. In either case, by comparing eligible schools to ineligible schools near the threshold, the RD estimator may provide a misleading description of the overall effects of the policy. This may be especially problematic for predicting the effects of the policy in later years because the schools near the eligibility have likely had fewer years of total eligibility than schools with higher poverty levels.

To test the representativeness of our RDD estimates, we compare the dynamic effects we estimate using the RD to instrument for cumulative eligibility with a difference-in-differences analysis of the introduction of the policy.^14^ Standard difference-in-differences models rely on ineligible schools as a control to estimate counterfactual time trends for the treated schools. The underlying assumption is that treated schools would have followed similar trends as untreated schools in the absence of the CSB policy. The substantial differences in the poverty rates between these groups makes this assumption problematic. We therefore conduct a difference-in-differences analysis using the sample of schools ever eligible for the bonus using data from 2003–2013:

(3) $$Y_{st}=X_{st}\beta +\text{NumYear}s_{\text{st}}\delta +\alpha_{s}+\lambda_{t}+\epsilon_{st}.$$

We then allow for nonlinear effects by estimating an event study with separate coefficients for each year relative to the first year of eligibility for the CSB:

(4) $$Y_{st}=X_{st}\beta +\Sigma_{\tau =-6\;}^{5}\text{FirstYea}r_{st+\tau }\delta_{\tau }+\alpha_{s}+\lambda_{t}+\epsilon_{st}.$$

**Fig. 5:**
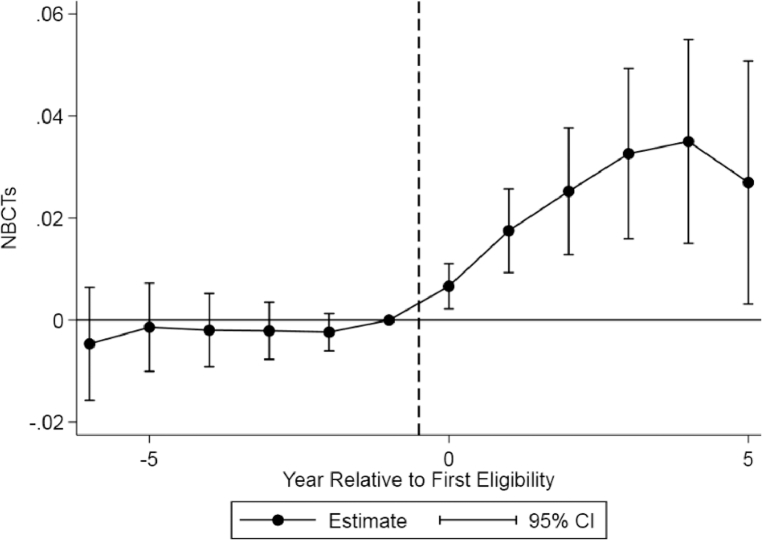
Event study of bonus introduction and school staffing. *Notes:* Event study estimates based on sample with FRL within 10 points of the eligibility thresholds. Estimates are relative to the first year a school is eligible. Covariates include student race/ethnicity, student FRL participation, and school year by school level effects. Standard errors clustered at the school level.

The identification of the dynamic effects therefore comes from variation in the timing of initial eligibility among ever-eligible schools.^15^ Using the specification in Eq. (3), we estimate that an additional year of eligibility increases the proportion of NBCTs by 0.0065 (standard error = 0.0019). This is about half the estimated effects of 0.0115 to 0.0164 from the RDD. To explore this discrepancy further, we plot coefficients from the event study specification in Fig. 5. The plot shows stable NBCT staffing levels before a school's initial eligibility followed by an increase immediately after the introduction of the bonus. The largest increase occurs in the second year after a school becomes eligible, although we find increases through the first five years of eligibility. Five years after a school's first eligibility, we estimate that the proportion of NBCTs has increased by 3.5 percentage points; this falls to 2.7 percentage points in the sixth year. The average effects are thus less than an extrapolation of the annual results from Table 6, which would indicate an increase of about 5.8–8.2 percentage points over the first five years. Although the graphical evidence in Fig. 3 suggests the effects may have been largest for schools near the eligibility threshold, the difference appears to result from the relatively higher weight put on earlier years of eligibility in the RDD. We estimate that schools just to the right of the eligibility threshold have had 1.94 years of cumulative eligibility on average and the results in Fig. 5 suggest that these years provide the largest effects on NBCT staffing levels. Even the effects shown in Fig. 5, however, represent an increase of about 45–60% in the number of NBCTs in high poverty schools over the first five to six years of eligibility.

### 4.3 Spillover effects of teacher bonus policies

The targeted bonuses increase pay for NBCTs, but only in certain schools. The program design may therefore negatively affect staffing in schools nearby eligible schools. Such effects would operate through the same channels as the positive effects on CSB staffing. For instance, some of the newly hired NBCTs in bonus-eligible schools may have left their previous schools in search of the bonus. But even if bonuses do not induce teachers to leave their schools, they may change the destination of mobile NBCTs. The policy may also lead teachers who are likely to become future NBCTs to sort into eligible schools. Each of these possibilities would reduce the number of NBCTs in ineligible schools near those eligible for the CSB.

To assess spillover effects, we estimate a difference-in-differences design similar to that in the prior section. As the main treatment variable, we use the proportion of teachers in other district schools that are eligible for the bonus. We construct this measure separately for each school level (elementary, middle, and high school) to account for the fact that bonus eligibility guidelines differ by school grade level and teacher credentials are often limited to a particular grade level. We also exclude a school's own status from the measure so that it is identified solely from eligibility in other district schools. As in the above analysis, we exclude high income districts that never have eligible schools. We then estimate

(5) $$Y_{sjdt}=X_{sjdt}\beta +\text{CS}B_{\text{sjdt}}\delta_{1}+\text{CSB}\_\text{Concentratio}n_{\text{jdt}}\delta_{2}+\alpha_{s}+\lambda_{jt}+\epsilon_{sjdt},$$

where *j* indexes school level and *d* indexes district.^16^ As with the analysis in Section 4.2, this design relies on changes in school staffing outcomes following the introduction of the bonus policy in nearby schools. In some models, we additionally include district-by-year effects, so that the concentration effect is identified based on changes in the number of eligible schools across different grade levels in the same district. This approach provides some guarantee that observed changes are not a consequence of district initiatives, such as additional stipends, release time for candidates, or loans for application fees, which may be associated with changes in the number of eligible schools.

The results for bonus eligibility, in Table 8, largely mirror the results from the RDD and prior DID models. Although our focus is on estimating changes in the flow of NBCTs to eligible and nearby schools, we do find that teachers in eligible schools are about 2.4 to 2.6 percentage points more likely to be NBCTs, which matches the RD estimates. Similarly, the DID estimates indicate that eligibility increases the rate at which NBCTs are hired and certified by about 1 percentage point each and reduces NBCT turnover by about 3 percentage points. On the other hand, we find mixed evidence of spillover effects on other district schools. In Panel A, we estimate that the marginal effect of additional CSB concentration on total NBCT staffing in other district schools is −0.02, which is statistically significant. However, the effect diminishes to −0.01, and is not significant, when we include district-by-year fixed effects in Panel B. In Panels C and D, we allow the effect of CSB concentration to differ between CSB-eligible and CSB-ineligible schools and find that any CSB effect appears to be concentrated among ineligible schools. The main effect for CSB concentration, −0.02, is significant and negative in Panel D with district-year fixed effects. The combined effect for CSB-eligible schools is near zero and statistically insignificant. Thus, ineligible schools in districts with higher concentrations of CSB eligible schools experienced reductions in their overall NBCT staffing levels. The average concentration among ineligible schools in districts with at least one CSB school was 0.38, which suggests a total reduction of about 0.2 NBCTs per ineligible school.

**Table 8:** Spillover effects of bonus eligibility.

	(1)	(2)	(3)	(4)
	NBCT	New hire	NBCT turnover	NB certification
*Panel A. District difference-in-differences estimates*				
CSB	0.0243⁎⁎⁎	0.0079⁎⁎	−0.0307	0.0095⁎⁎⁎
	(0.0047)	(0.0034)	(0.0194)	(0.0010)
CSB Concentration	−0.0223⁎⁎	−0.0098	−0.0081	−0.0065⁎⁎
	(0.0085)	(0.0072)	(0.0265)	(0.0025)
N	8,600	8,132	2,996	8,600
*Panel B. District-by-year fixed effects estimates*				
CSB	0.0262⁎⁎⁎	0.0111⁎⁎⁎	−0.0260	0.0110⁎⁎⁎
	(0.0051)	(0.0041)	(0.0206)	(0.0013)
CSB Concentration	−0.0132	0.0055	−0.0208	−0.0019
	(0.0095)	(0.0087)	(0.0414)	(0.0039)
N	8,569	8,081	2,838	8,569
*Panel C. Interaction effects (District DID)*				
CSB	0.0288⁎⁎⁎	0.0166⁎⁎⁎	−0.0370	0.0130⁎⁎⁎
	(0.0068)	(0.0059)	(0.0276)	(0.0020)
CSB Concentration	−0.0171*	0.0013	−0.0140	−0.0025
	(0.0094)	(0.0094)	(0.0327)	(0.0030)
CSB x CSB concentration	−0.0121	−0.0244*	0.0159	−0.0094⁎⁎
	(0.0118)	(0.0126)	(0.0441)	(0.0045)
N	8,600	8,132	2,996	8,600
*Panel D. Interaction effects (District-by-Year Effects)*				
CSB	0.0202⁎⁎⁎	0.0126*	−0.0288	0.0119⁎⁎⁎
	(0.0072)	(0.0073)	(0.0317)	(0.0023)
CSB concentration	−0.0186⁎⁎	0.0070	−0.0229	−0.0012
	(0.0086)	(0.0095)	(0.0460)	(0.0040)
CSB x CSB concentration	0.0181	−0.0047	0.0078	−0.0025
	(0.0166)	(0.0158)	(0.0671)	(0.0063)
N	8,569	8,081	2,838	8,569

*Notes:* Difference-in-differences estimates of CSB and CSB concentration effects estimated with observation weights. CSB concentration indicates the proportion of teachers in other district schools working in CSB-eligible schools. Covariates include school demographics and enrollment interacted with school level and level-by-year fixed effects. Models in Panel B additionally include district-by-year fixed effects. Models in Panels C and D additionally add interactions between a school's CSB status and the CSB concentration measure. Standard errors clustered by district are in parentheses.

⁎ *p* < 0.10,

⁎⁎ *p* < 0.05,

⁎⁎⁎ *p* < 0.01.

We find inconsistent evidence on specific mechanisms by which the bonus reduces the proportion of NBCTs in other district schools. Although the coefficients on the rate of newly hired NBCTs and new certifications are in the expected direction in the difference-in-differences models in Panel A, only the coefficient on new certification is statistically significant. We estimate that having nearby eligible schools actually reduces NBCT turnover, although the estimate is not statistically significant. In Panel B, with district-by-year effects, none of the coefficients on district concentration is significant. Consistent with these findings, we do not estimate significant effects of CSB concentration among ineligible schools in Panels C or D. Taken together, the results do not provide clear evidence of effects of targeted bonuses on staffing in nearby schools, although they do appear to affect staffing in ineligible schools. This finding may be dependent on the design of the CSB policy. The policy targets a credential that any teacher could hypothetically earn, and we find that much of the effect of the bonus on a school's workforce is driven by increases in certification rates among incumbent teachers. Thus, the effect of the bonus need not be entirely offset by reductions in NBCTs in other schools. In particular, the policy does not appear to induce mobility among NBCTs in neighboring schools.

Readers may be concerned that the potential for negative spillover effects on ineligible district schools biased upward our RD estimates above. In particular, if the bonus induces teachers to move from eligible to ineligible schools, the changes in NBCT staffing at the eligibility threshold may over count the increase in NBCTs among eligible schools. Although we do not find evidence that the bonus affects NBCT turnover in ineligible schools, we do find that having more eligible schools in a district reduces NBCT staffing for ineligible schools. Nonetheless, the left limit on the concentration measure among ineligible schools at the eligibility threshold is only 0.06, suggesting an upper bound on the potential spillover bias of 0.001.^17^

## 5 Additional robustness checks

In Table 9, we conduct a number of sensitivity tests of the RDD. In the first three columns, we test the specification of the relationship between the forcing variable and NBCT staffing outcomes by implementing the RDD using different bandwidths. Each entry displays the coefficient on the cumulative years of CSB eligibility. In columns (1) and (2), we use half and twice of the Imbens and Kalyanaraman (2012) optimal bandwidth. The estimated effects are generally similar to main results. Using half the optimal bandwidth, the NBCT effect is not statistically significant without covariates, but the point estimate for an additional year of eligibility (0.009) is similar in magnitude to the estimate in Table 6. The point estimate with covariates (0.014) is statistically significant. At twice the optimal bandwidth, the point estimates (0.010 and 0.015) are statistically significant and again similar to the results in Table 6.^18^ Finally, we test the sensitivity of our results to the specification of the first-stage regression. In the baseline regressions, we scale each of the discontinuities in outcomes by the discontinuity in the number of years of eligibility and use the optimal bandwidth for the outcome variable. In column (3), we use the optimal bandwidth for the treatment variable instead. The point estimate without covariates (0.009) is only statistically significant at the 10% level, although the estimate with covariates (0.015) is statistically significant. Finally, in column (4) we use the optimal bandwidth for each stage of the IV regression separately and estimate the effect of an additional year of eligibility as the ratio of the two discontinuities. The plot of eligibility and school FRL in Fig. 2 indicates that we may overestimate the change in the probability of treatment at the discontinuity, which would tend to depress our estimates of the CSB effect. Although the point estimates (0.014 and 0.021) are slightly larger than the baseline estimates, the magnitude is similar and both pairs of coefficients are statistically significant.

**Table 9:** Sensitivity Tests for the Regression Discontinuity Design.

	(1)	(2)	(3)	(4)	(5)	(6)
Baseline RDD	0.0088	0.0102⁎⁎⁎	0.0092*	0.0142⁎⁎⁎	0.0125⁎⁎⁎	0.0138⁎⁎⁎
	(0.0062)	(0.0030)	(0.0051)	(0.0053)	(0.0039)	(0.0041)
N	3,662	10,119	4,686	6,877	6,767	6,415
RDD with covariates	0.0144⁎⁎⁎	0.0147⁎⁎⁎	0.0153⁎⁎⁎	0.0206⁎⁎⁎	0.0175⁎⁎⁎	0.0185⁎⁎⁎
	(0.0052)	(0.0025)	(0.0042)	(0.0043)	(0.0032)	(0.0035)
N	3,613	10,000	4,621	6,789	6,679	6,330
Bandwidth	0.5x (0.17)	2x (0.68)	FS (0.22)	Separate	>0.5pt	>2pt

*Notes:* The regression discontinuity models are estimated by two-stage least squares with triangular kernel and observation weights. The treatment variable in each model is the cumulative number of years of CSB eligibility. The models in columns (1) and (2) use half and twice the Imbens and Kalyanaraman (2012) optimal bandwidth, respectively. The models in column (3) use the optimal bandwidth for the treatment variable rather than the outcome variable. The estimates in column (4) estimate the first and second stage using optimal bandwidths for each stage (0.22 for the first stage and 0.34 for the second stage). The estimates in columns (5) and (6) are from “donut” regression discontinuity models that omit observations within 0.5 and 2 percentage points of the eligibility threshold, respectively. Covariates include 2007 school values of the proportion of NBCTs, proportion of NBCT applicants, average teacher experience, average teacher educational attainment, average teacher turnover, math and reading achievement, student race/ethnicity, student FRL participation, and school year and school level effects. Standard errors clustered by school are in parentheses. Standard errors clustered by school are in parentheses. Standard errors in column (4) estimated by school-clustered bootstrap with 399 iterations.

⁎ *p* < 0.10, ^⁎⁎^*p* < 0.05,

⁎⁎⁎ *p* < 0.01.

As we discuss above, the main empirical findings are robust to the inclusion of pre-treatment outcome variables. However, the discontinuities in lagged outcomes and student characteristics suggest caution about interpreting the results as causal. In the remaining columns, we provide further tests of whether manipulation in the forcing variable may be driving our results. Our primary concern is that schools may be able to perturb their FRL enrollment counts in order to become just eligible for the CSB. If this kind of manipulation is associated with potential outcomes, then the RDD is no longer valid. We therefore follow Barreca, Guldie, Lindo, and Waddell (2011) and re-estimate the models after omitting schools closest to the eligibility threshold. This approach should remove potential manipulators, but relies on an extrapolation based on schools farther from the discontinuity. We omit schools within 0.5 percentage points of the eligibility threshold in columns (5) schools within 2 percentage points in column (6). In both cases, the estimates are similar to the main results in Table 6. Consistent with the negative sorting on baseline outcomes apparent in Table 5, the point estimates are actually somewhat larger when omitting schools near the threshold. It does not appear that manipulation in the forcing variable significantly influences our results.

## 6 Certification bonuses and the signaling value of credentials

One potential difference between the CSB and other targeted compensation policies is that mid-career teachers can relatively easily earn certification. The incentive to earn certification could plausibly lead the CSB policy to affect the underlying relationship between student achievement and the targeted credential. If we assume that certification is more costly for less effective teachers, which seems reasonable given the positive correlation between assessment scores and teacher value added indicated by Cantrell, Fullerton, Kane, and Staiger (2008) and Cowan and Goldhaber (2016), then a signaling argument would suggest that newly certified teachers were on average less effective than those earning the certificate in the absence of additional bonuses. That is, we would expect that teachers on the margin of seeking certification were less effective on average than those who would otherwise have obtained the credential. If this were the case, then the bonus policy may have partially undermined the usefulness of professional certification as a tool for attracting effective teachers to high poverty schools. This may provide another explanation for the null findings of the effects of the policy on student achievement.

The student achievement data provides a direct measure of teacher productivity. We can therefore test whether teachers who earned certification in schools eligible for the CSB were less effective than those who earned certification in ineligible schools. To do so, we use a sample of students matched to classroom teachers in elementary and middle school grades.^19^ We first compare the effectiveness of teachers earning certification while teaching in a school eligible for the bonus to other certified teachers. We estimate

(6) $$Y_{ijst}=X_{ijst}\beta +NBCT_{jt}\delta_{1}+NBCT_{jt}\times CertCSB_{j}\delta_{2}+\alpha_{st}+\epsilon_{ijst}$$

where *X* is a vector of student characteristics, including lagged test scores, *NBCT* indicates that a teacher earns certification in our sample period, *CertCSB* indicates that the teacher earned certification while her school was eligible for the CSB, and α is a school-by-year fixed effect. This model is mainly descriptive in that it makes no attempt to control for differences in the effectiveness of teachers earning certification at different types of schools nor adjust for any differences in selectivity across certification tests or time. To provide a better sense of whether eligibility influences the pool of certified teachers, we estimate a difference-in-differences type specification of Eq. (6) that additionally adds an indicator for earning certification in a school that is ever eligible for the CSB and either an indicator for earning certification after 2008 (the first year of the CSB program) or certification test-by-cohort fixed effects.

The results of these regressions, in Table 10, exhibit no evidence that the certification bonus reduced the effectiveness of newly certified teachers. The coefficients on NBCT status in the first row of each panel indicate that students of National Board certified teachers perform about 0.02–0.03 standard deviations higher on state assessments in math and reading, depending on the model. These results are consistent with prior research on NBCTs (Cantrell et al., 2008, Clotfelter et al., 2007, Cowan and Goldhaber, 2016, Goldhaber and Anthony, 2007). We do not find that teachers certified in CSB-eligible schools are less effective than those earning certification either before the program began or in ineligible schools; however, the point estimates are positive and statistically significant for math in models without school fixed effects. Further, it does not appear that eligibility for the CSB changes the effectiveness of teachers earning certification. The difference-in-differences models in columns (3) through (5) find no reduction in the effectiveness of NBCTs in eligible schools (indicated by the coefficient on NBCT, eligible school).

**Table 10:** The effectiveness of teachers certified with bonus eligibility.

	(1)	(2)	(3)	(4)	(5)
*Panel A. Math teachers*					
NBCT	0.0332⁎⁎⁎	0.0340⁎⁎⁎	0.0156	0.0200	
	(0.0076)	(0.0067)	(0.0124)	(0.0115)	
NBCT, eligible school	0.0458⁎⁎	0.0149	0.0285	0.0093	0.0266
	(0.0176)	(0.0144)	(0.0250)	(0.0210)	(0.0213)
NBCT, ever-eligible school			0.0106	−0.0009	−0.0130
			(0.0221)	(0.0188)	(0.0189)
NBCT, post-2008			0.0245	0.0202	
			(0.0154)	(0.0136)	
*N*	1,312,566	1,312,566	1,312,566	1,312,566	1,312,566
*Panel B. Reading teachers*					
NBCT	0.0250⁎⁎⁎	0.0174⁎⁎⁎	0.0294⁎⁎	0.0107	
	(0.0050)	(0.0047)	(0.0089)	(0.0088)	
NBCT, eligible school	0.0031	0.0051	0.0094	0.0067	0.0072
	(0.0121)	(0.0114)	(0.0174)	(0.0172)	(0.0174)
NBCT, ever-eligible school			−0.0052	−0.0054	−0.0064
			(0.0150)	(0.0149)	(0.0149)
NBCT, post-2008			−0.0056	0.0104	
			(0.0107)	(0.0101)	
*N*	1,234,910	1,234,910	1,234,910	1,234,910	1,234,910
School-by-year FE	N	Y	N	Y	Y
Certification test-by-cohort FE	N	N	N	N	Y

*Notes:* Estimates are from student-level data over the period 2007–2013. The sample is described in Cowan and Goldhaber (2016). All models include controls for cubic polynomials in lagged achievement, student race/ethnicity, subsidized lunch status, participation in special education, bilingual education, and gifted education services, and the classroom means of these variables. “NBCT” indicates the teacher is board certified, “NBCT, eligible school” indicates the teacher is board certified and earned certification in a school currently eligible for the CSB, “NBCT, ever-eligible school” indicates that the teacher earned certification in a school that is ever eligible for the CSB, and “NBCT, post-2008″ indicates that the teacher earned certification in 2008 or after. Certification test-by-cohort fixed effects are defined as unique combinations of the certificate type and year. Standard errors clustered by school are in parentheses.

*p < 0.10,

⁎⁎ *p* < 0.05,

⁎⁎⁎ *p* < 0.01.

## 7 Conclusion

We study the introduction of an incentive for National Board certified teachers to work in high poverty schools and find that the bonus increased the proportion of teachers with the professional certificates. Depending on the method of analysis, we estimate that after five years of eligibility for the bonus, the percentage of board certified teachers would have increased by about four to eight percentage points. As in other studies of differentiated compensation for high needs schools, we find a reduction in the turnover rates for affected teachers. However, the bonus program also appears to have changed the characteristics of newly hired teachers. Although the findings of Protik et al. (2015) suggest that transfer bonus policies may not induce large numbers of teachers to switch schools, our results indicate that they may affect the composition of a school's applicant pool. About half of the increase is explained by teachers in eligible schools becoming certified. Although eligibility for the bonus increased the likelihood that incumbent teachers apply for certification, we find little evidence that the introduction of the bonus policy diluted the signaling value of the certificate. Teachers credentialed in high poverty schools following the introduction of the bonus are at least as effective as those credentialed beforehand.

Importantly, however, the increase in the number of NBCTs does not appear to have led to detectible improvements in student achievement, a finding which is consistent with the magnitude of staffing changes we observe as well as the prior evidence on the instructional effects of NBCTs. Nonetheless, it is possible that the CSB policy may have influenced student outcomes through other mechanisms. There is little direct evidence for NBCTs’ effects on non-tested student outcomes, but Gershenson (2016) and Jackson (2016) suggest that achievement gains may be poor predictors of other dimensions of teacher effectiveness. Instead, the strength of the relationship between student outcomes and observable teacher characteristics may be a limiting factor for differentiated compensation policies based on teacher credentials. Although several prior studies have found that NBCTs are more effective in the classroom, observable credentials explain little of the variation in teachers’ contributions to student achievement. The achievement findings from this study may therefore not generalize to policies that use direct performance measures, such as teacher evaluations, to target incentives (e.g., Adnot, Dee, Katz, & Wyckoff, 2017).

## Appendix Supplementary materials

Image, application 1

## Footnotes

1 The NBPTS offers a national, voluntary certification process consisting of a yearlong sequence of assessments. Successful completion of the certification process is meant to indicate that a teacher has accomplished a high level of practice akin to board certification in medicine. Nationally, about 3% of teachers have earned NBPTS certification (Exstrom, 2011). The NBPTS credential is also commonly used by states as a signal of teacher quality. A majority of states accept NBPTS certification as a means of satisfying state licensing requirements and provide some financial incentive for teachers to earn certification (Exstrom, 2011, National Board for Professional Teaching Standards 2015).

2 We describe a few studies that examine recruitment below; however, each of these papers examines the effects of signing bonuses rather than policies that award permanent, but generally smaller, salary increases.

3 The amount of the bonus is prorated by the proportion of time teachers spend in an eligible school. Starting with the 2011–2012 school year, the amount of the bonus was reduced to $3,000 for the first year a teacher earns National Board certification.

4 The Comprehensive Education Data and Research System (CEDARS) replaced CSRS in 2010. We refer to this as the “CSRS” measure throughout for simplicity.

5 About 18% of all teaching staff reports a position in more than one building. However, this includes teachers with an assignment reported at the district office and may overstate the number of teachers who work in more than one school.

6 We omit schools that ever enroll fewer than 30 students, which are generally not eligible to qualify for the CSB, and schools missing enrollment information required for determination of eligibility. We also omit schools with no K-12 enrollment (2 schools) or teacher staffing information (17 schools) reported in any year. In addition, in the eligibility data provided by OSPI, there were three schools whose reported CSB status in one year did not correspond either to the reported FRL enrollment data or to eligibility reports from other years. Because we could not verify with OSPI whether these schools actually received the bonus, we dropped them from our study. The final sample includes 1,698 schools, or 98.7% of the schools subject to the eligibility determination for the CSB.

7 The S-275 includes all teachers employed by October 1 of the reporting year, so our sample may exclude a small number of teachers who are laid off and subsequently recalled or who sign a contract after October 1 and stay at the school for only one year. Given that teachers are only eligible for National Board certification if they have more than 3 years of experience, this form of measurement error is unlikely to substantially affect our measurement of NBCTs.

8 We implement the density test using the Stata package DCdensity McCrary, 2008a, McCrary, 2008b). Given the grouped nature of the data, we estimate standard errors using a clustered bootstrap on schools with 400 iterations.

9 In the baseline models, we do not include any additional variables (such as grade level of year effects). Although the policy rule differs by school level and year, their inclusion should only affect the results if there are differences in the likelihood that schools are just-eligible or just-ineligible across grades and years. Regardless, their inclusion results in nearly identical point estimates.

10 We assume a discount rate of 3% and that the probability of future eligibility for schools just missing eligibility in the current year is 0.5.

11 Visual inspection of Fig. 2 suggests that the local linear regressions fit the data poorly for schools in the bin just to the left of the discontinuity. We revisit this issue in Section 5 and show that the results are not particularly sensitive to using a narrower bandwidth for the first stage regression.

12 The Institute of Education Sciences (2017) recommends examining the discontinuities in the standardized outcomes and including adjustments for any baseline measure with a discontinuity larger than 0.05 standard deviations. They further consider any discontinuity greater than 0.25 standard deviations reason for significant concern about the integrity of the research design. None of the discontinuities in the baseline measures in Table 5 is larger than 0.25 standard deviations. Following this guidance, we show that the results are robust to the inclusion of the baseline covariates, some of which have discontinuities greater than 0.05 standard deviations.

13 These figures use the estimates from the specifications with covariates. We find very similar results using models without covariates, although they explain about 90 percent of the estimated annual effect from Table 6.

14 An alternative to our difference-in-differences approach is to estimate a dynamic regression discontinuity model in the spirit of the one-step estimator of Cellini, Ferreira, and Rothstein (2010) that treats each lag of program eligibility as a separate RD. We do not pursue this approach here. In this context, the grandfathering provisions of the CSB policy and high re-enrollment rates for FRL programs ensure that there is high autocorrelation in both observed treatment and FRL enrollment share. Given that this method identifies lagged treatment effects by comparing schools with similar observed FRL histories but different patterns of treatment, estimates using this approach are very imprecise. Further, part of our interest in estimating alternative specifications is comparing the LATE estimated from an RDD to treatment effects estimated for a lager set of schools.

15 As with the analysis of the RDD above, we uncover little evidence of a detectible effect on student achievement. The results (available from the authors on request) are similar in magnitude and not statistically significant. There is also little consistent evidence of an effect from comparisons of mean test scores before and after the introduction of the policy.

16 Recall that mobility is defined as whether a teacher returns in the following school year and that we use a lead of the CSB policy indicator to match end-of-year retention decisions with the job opportunities in the upcoming school year.

17 We compute this by multiplying the limit on CSB concentration by the effect estimated in Panel D for ineligible schools. Note that the bias will be less than this since not all schools at the threshold are ineligible.

18 As an additional test of the specification of the local linear regressions, Imbens and Lemieux (2008) recommend testing for discontinuities in the outcome variables at the median points of the forcing variable above and below the eligibility threshold. Neither of the estimated discontinuities is statistically significant at any conventional level (results available from the authors upon request).

19 See Cowan and Goldhaber (2016) for a description of the sample used in this analysis.

## References

[bib0001] Aaronson D., Barrow L., Sander W. (2007). Teachers and student achievement in the Chicago Public High Schools. Journal of Labor Economics.

[bib0002] Adnot M., Dee T., Katz V., Wyckoff J. (2017). Teacher turnover, teacher quality, and student achievement in DCPS. Educational Evaluation and Policy Analysis.

[bib0003] American Federation of Teachers & National Education Association (2007). A guide to understanding national board certification: 2008–2009 candidacy cycle.

[bib0004] American Federation of Teachers & National Education Association (2008). A guide to understanding national board certification: 2009-10 candidacy cycle.

[bib0005] Barreca A.I., Guldie M., Lindo J.M., Waddell G.R. (2011). Saving babies? Revisiting the effect of very low birthweight classification. Quarterly Journal of Economics.

[bib0006] Cantrell S., Fullerton J., Kane T.J., Staiger D.O. (2008). National board certification and teacher effectiveness: Evidence from a random assignment experiment (No. 14608).

[bib0007] Cavalluzzo L., Barrow L., Henderson S., Mokher C., Geraghty T., Sartain L. (2015). From large urban to small rural schools: An empirical study of national board certification and teaching effectiveness.

[bib0008] Cellini S.R., Ferreira F., Rothstein J. (2010). The value of school facility investments: Evidence from a dynamic regression discontinuity design. Quarterly Journal of Economics.

[bib0009] Chetty R., Friedman J.N., Rockoff J.E. (2014). Measuring the impacts of teachers II: Teacher value-added and student outcomes in adulthood. American Economic Review.

[bib0010] Chingos M.M., Peterson P.E. (2011). It's easier to pick a good teacher than to train one: Familiar and new results on the correlates of teacher effectiveness. Economics of Education Review.

[bib0011] Clotfelter C.T., Glennie E., Ladd H., Vigdor J. (2008). Would higher salaries keep teachers in high poverty schools? Evidence from a policy intervention in North Carolina. Journal of Public Economics.

[bib0012] Clotfelter C.T., Ladd H.F., Vigdor J. (2005). Who teaches whom? Race and the distribution of novice teachers. Economics of Education Review.

[bib0013] Clotfelter C.T., Ladd H.F., Vigdor J. (2007). Teacher credentials and student achievement: Longitudinal analysis with student fixed effects. Economics of Education Review.

[bib0014] Clotfelter C.T., Ladd H.F., Vigdor J. (2010). Teacher credentials and student achievement in high school: A cross-subject analysis with student fixed effects. Journal of Human Resources.

[bib0015] Cowan J., Goldhaber D. (2016). National Board certification and teacher effectiveness: Evidence from Washington State. Journal of Research on Educational Effectiveness.

[bib0016] Elfers A.M., Plecki M.L. (2014). Results of a state incentive program on the supply and distribution of National Board certified teachers. Leadership and Policy in Schools.

[bib0017] Exstrom M. (2011). National board for professional teaching standards certification: What legislators need to know.

[bib0018] Feng L., Sass T. (2017). The impact of incentives to recruit and retain teachers in “hard-to-staff” subjects. Educational evaluation and policy analysis.

[bib0019] Field E. (2008). Educational debt burden and career choice: Evidence from a financial aid experiment at NYU Law School. American Economic Journal: Applied Economics.

[bib0020] Gershenson S. (2016). Linking teacher quality, student attendance, and student achievement. Education Finance and Policy.

[bib0021] Glazerman S., Protik A., Teh B.-R., Bruch J., Max J. (2013). Transfer incentives for high-performing teachers: Final results from a multisite randomized experiment (No. 2014-4003). National center for education evaluation and regional assistance.

[bib0022] Goldhaber D., Anthony E. (2007). Can teacher quality be effectively assessed? National Board certification as a signal of effective teaching. Review of Economics and Statistics.

[bib0023] Goldhaber D., Choi H.-J., Cramer L. (2007). A descriptive analysis of the distribution of NBPTS-certified teachers in North Carolina. Economics of Education Review.

[bib0024] Goldhaber D., Hansen M. (2009). National Board certification and teachers’ career paths: Does NBPTS certification influence how long teachers remain in the profession and where they teach?. Education Finance and Policy.

[bib0025] Goldhaber D., Lavery L., Theobald R. (2015). Uneven playing field? Assessing the teacher quality gap between advantaged and disadvantaged students. Educational Researcher.

[bib0026] Goldhaber D., Liddle S., Theobald R., Walch J. (2012). Teacher effectiveness and the achievement of Washington's students in mathematics. WERA Educational Journal.

[bib0027] Goldhaber D., Strunk K.O., Brown N., Knight D.S. (2016). Lessons learned from the Great Recession: Layoffs and the RIF-induced teacher shuffle. Educational Evaluation and Policy Analysis.

[bib0028] Hanushek E.A., Kain J.F., Rivkin S.G. (2004). Why public schools lose teachers. Journal of Human Resources.

[bib0100] Harris D.N., Sass T.R. (2009). The effects of NBPTS-certified teachers on student achievement. Journal of Policy Analysis and Management: [the Journal of the Association for Public Policy Analysis and Management].

[bib0029] Harris D.N., Sass T.R. (2011). Teacher training, teacher quality and student achievement. Journal of Public Economics.

[bib0030] Hendricks M.D. (2014). Does it pay to pay teachers more? Evidence from Texas. Journal of Public Economics.

[bib0031] Humphrey D.C., Koppich J.E., Hough H.J. (2005). Sharing the wealth: National Board certified teachers and the students who need them most. Education Policy Analysis Archives.

[bib0032] Imazeki J. (2005). Teacher salaries and teacher attrition. Economics of Education Review.

[bib0033] Imbens G., Kalyanaraman K. (2012). Optimal bandwidth choice for the regression discontinuity estimator. Review of Economic Studies.

[bib0101] Imbens G.W., Lemieux T. (2008). Regression discontinuity designs: A guide to practice. Journal of Econometrics.

[bib0034] Institute of Education Sciences (2017). What works clearinghouse standards handbook, version 4.

[bib0035] Jackson C.K. (2016). What do test scores miss? The importance of teacher effects on non-test score outcomes (No. 22226).

[bib0036] Klein A. (2015). Ed. dept. approves teacher-equity plans for 16 states. Education Week.

[bib0037] Klein A. (2015). States struggle with how to ensure good teachers in all schools. Education Week.

[bib0038] Klein A. 2015c U.S. department of education approves more state teacher-distribution plans http://blogs.edweek.org/edweek/campaign-k-12/2015/10/us_department_of_education_app.html?cmp=SOC-SHR-FB Accessed: 2015-11-10.

[bib0039] Koedel C. (2008). Teacher quality and dropout outcomes in a large, urban school district. Journal of Urban Economics.

[bib0040] Lankford H., Loeb S., Wyckoff J. (2002). Teacher sorting and the plight of urban schools: A descriptive analysis. Educational Evaluation and Policy Analysis.

[bib0041] Mansfield R.K. (2015). Teacher quality and student inequality. Journal of Labor Economics.

[bib0042] Matsudaira J.D., Hosek A., Walsh E. (2012). An integrated assessment of the effects of Title I on school behavior, resources, and student achievement. Economics of Education Review.

[bib0043] McCrary, J. (2008a). DCdensity [Stata program]. Retrieved from https://eml.berkeley.edu/~jmccrary/DCdensity.

[bib0044] McCrary J. (2008). Manipulation of the running variable in the regression discontinuity design: A density test. Journal of Econometrics.

[bib0046] National Board for Professional Teaching Standards (2010). 2011 guide to national board certification.

[bib0047] National Board for Professional Teaching Standards (2010). Profiles in excellence: Washington state.

[bib0048] National Board for Professional Teaching Standards (2014). 2014 state rankings by new number of national board certified teachers.

[bib0049] National Board for Professional Teaching Standards (2014). 2014 state rankings by total number of national board certified teachers.

[bib0050] National Board for Professional Teaching Standards (2015). State incentive chart.

[bib0051] National Center for Education Statistics (2015). Schools and staffing survey, public school teachers: 2011-2012.

[bib0052] Nye B., Konstantopoulos S., Hedges L.V. (2004). How large are teacher effects?. Educational Evaluation and Policy Analysis.

[bib0053] Office of the Superintendent of Public Instruction (2014). 2014 proviso reports: NBPTS cert. Salary bonuses.

[bib0054] Pathman D.E., Konrad T.R., King T.S., Taylor D.H., Koch G.G. (2004). Outcomes of states’ scholarship, loan repayment, and related programs for physicians. Medical Care.

[bib0055] Plecki M.L., Elfers A.M., St. John E., Finster M., Emry T., Nishida N. (2010). Study of the incentive program for Washington's National Board certified teachers.

[bib0056] Protik A., Glazerman S., Bruch J., Teh B.-R. (2015). Staffing a low-performing school: Behavioral responses to selective teacher transfer incentives. Education Finance and Policy.

[bib0057] Rabinowitz H.K., Diamond J.J., Markham F.W., Hazelwood C.E. (1999). A program to increase the number of family physicians in rural and underserved areas: Impact after 22 years. Journal of the American Medical Association.

[bib0058] Rivkin S.G., Hanushek E.A., Kain J.F. (2005). Teachers, schools, and academic achievement. Econometrica.

[bib0059] Rosen S., Ashenfelter O., Layard R. (1986). The theory of equalizing differences.

[bib0060] Sass T.R., Hannaway J., Xu Z., Figlio D.N., Feng L. (2012). Value added of teachers in high poverty schools and lower poverty schools. Journal of Urban Economics.

[bib0061] Sato M., Wei R.C., Darling-Hammond L. (2008). Improving teachers’ assessment practices through professional development: The case of National Board certification. American Educational Research Journal.

[bib0062] Simpkins J. (2011). What does washington state get for its investment in bonuses for board certified teachers?.

[bib0064] Springer M.G., Swain W.A., Rodriguez L.A. (2016). Effective teacher retention bonuses: Evidence from Tennessee. Educational Evaluation and Policy Analysis.

[bib0065] Steele J.L., Murnane R.J., Willett J.B. (2010). Do financial incentives help low-performing schools attract and keep academically talented teachers? Evidence from California. Journal of Policy Analysis and Management.

[bib0066] Ushomirsky N., Williams D. (2015). Funding gaps 2015. The Education Trust.

[bib0067] Williams W., Adrien R., Murthy C., Pietryka D. (2016). Equitable access to excellent educators: An analysis of states’ educator equity plans.

